# The myeloid SRC family kinase HCK regulates breast cancer growth by activating tumor-associated macrophage-led invasion and inhibiting cytotoxic T cell activity

**DOI:** 10.3389/fimmu.2026.1709102

**Published:** 2026-02-18

**Authors:** Michael W. Murrey, Ashleigh R. Poh, James H. Steer, Catherine Rinaldi, Kellie A. Mouchemore, Amy R. Dwyer, Elena Denisenko, Irina Kuznetsova, Yen Yeow, Matthew E. Jones, Khaing P. W. Hmon, Dáithí Ó Muirí, Ya-Yu Liu, Weitao Lin, Alistair R. R. Forrest, Lesley G. Ellies, David A. Joyce, Matthias Ernst, Fiona J. Pixley

**Affiliations:** 1 School of Biomedical Sciences, The University of Western Australia, Crawley, WA, Australia; 2 Olivia Newton-John Cancer Research Institute and School of Cancer Medicine, La Trobe University, Heidelberg, VIC, Australia; 3 Centre for Microscopy, Characterisation and Analysis, The University of Western Australia, Crawley, WA, Australia; 4 Harry Perkins Institute of Medical Research, Nedlands, WA, Australia

**Keywords:** breast cancer, cytotoxic T cells, HCK, tumor invasion, tumor-associated macrophages

## Abstract

**Introduction:**

The normal developmental and homeostatic roles of tissue resident macrophages are subverted in tumor-associated macrophages to promote tumor progression. Pro-tumoral macrophage activities include immune suppression and promotion of invasion and metastasis. While the myeloid Src family kinase HCK is known to regulate immune evasion, here we show that HCK promotes growth of an aggressively invasive mammary tumor through activation of macrophage motility and invasive capacity.

**Method:**

We used the Py8119 mouse mammary tumor model to investigate the role of Hck activity in tumor growth through therapeutic inhibition and genetic modification. Single cell RNA sequencing and immunohistochemistry approaches were used to investigate changes to the immune compartment.

**Results:**

Loss of HCK activity reduced the growth of Py8119 mammary tumors by 70-80% while excessive HCK activity increased growth four-fold. Consistent with a role for HCK in macrophage invasiveness, plasma membrane-associated Src family kinase activity at the tumor margins was lost in the absence of HCK. Regarding immune evasion, HCK-deficient tumors contained increased CD8^+^ T cell numbers and, while CD8+ T cell depletion reduced survival in all mouse cohorts, CD8^+^ T cell-depleted HCK-deficient mice survived much longer than CD8^+^ T cell-replete control mice. Characterisation of the tumour microenvironment by single cell sequencing showed 5 major subtypes of TAMs (immunoregulatory (Folr2^high ^), inflammatory (MHC-II^high ^), interferon-primed, angiogenic and tissue resident), which together made up 40% of cells in the tumour. While Hck activity did not affect the recruitment of TAMs or their subtype composition, pathway analysis showed that its loss decreased TAM motility and increased their interferon signalling and reduced EMT pathways in the Py8119 tumor cells.

**Conclusion:**

This study provides evidence that HCK activity in TAMs enhances tumour growth via promotion of invasive behaviour as well as suppression of anti-tumor immunity. These findings highlight HCK as a promising therapeutic target to limit tumor progression.

## Introduction

Cells of a myeloid origin, particularly tumor associated macrophages (TAMs), play important roles as determinants of tumor progression and response to therapy in all solid malignancies. Macrophage functional plasticity underpins the ability of TAMs to orchestrate complex and variable responses to tumors as they progress (1). Accordingly, the effects of the local microenvironment on TAMs have been linked to the functional dichotomy between the conventional activation of macrophages to execute their normal homeostatic activities in tissues and the various tumor promoting TAM endotypes or subtypes that arise from alternative activation cues in tumors (2). Indeed, the latter encompass different functional subsets including those with angiogenic, immune-suppressive, inflammatory and interferon-responsive characteristics (3, 4). Collectively however, TAMs retain the capacity for interstitial migration, normally required by tissue resident macrophages for development and homeostasis, including guiding the formation of the mammary gland ductal network (5, 6). These insights suggest that TAMs may serve as therapeutic targets to help control tumor promotion by either limiting their abundance in the tumor microenvironment, suppressing their activation toward alternatively activated endotypes or blocking a specific tumor-promoting behavior.

Therapeutic control of macrophage activation and associated endotypes has become a major interest for the control of cancer. Several molecules have been identified that appear to play a role at the apex of signaling cascades controlling TAM plasticity and in particular limiting and/or reverting alternative activation. Among those, inhibition of phosphatidyl inositol 3-kinase (PI3K)γ or of the myeloid Src family kinase (SFK) hematopoietic cell kinase (HCK) has attracted interest to convert immune-suppressive to immune-permissive tumor microenvironments (7, 8). Interestingly, both PI3K p110δ and HCK are functionally associated with the receptor for colony-stimulating factor-1 (CSF-1R), which not only promotes mononuclear phagocyte maturation along the monocyte-macrophage trajectory but also regulates macrophage motility and extracellular matrix (ECM) remodeling (9–11). Indeed, selective inhibition of either p110δ or HCK impairs macrophage motility *in vitro* (9). Furthermore, Py8119 mammary tumor spheroids require direct contact with pre-infiltrated bone marrow-derived macrophages (BMM) to invade surrounding Matrigel, which is blocked by HCK inhibition whereas BMM that express a constitutively active isoform of HCK (*Hck^CA^*) are hyper-motile and promote two-fold increased invasion of tumor cells into the surrounding matrix (12, 13). TAMs derived from tumor-bearing *Hck^CA^* hosts also show a pronounced bias towards alternative activation, thereby limiting the anti-tumor immune responses conferred by CD8 and NK effector cells across models of colon, gastric and pancreatic cancer (13–15). This occurs despite myeloid cells contributing a relatively low proportion of cells in these tumors and correlates with suppression of effector cell infiltration and an associated reduction of effector molecules alongside excessive extracellular matrix deposition.

In this study, we show that ablation of HCK activity in a myeloid cell-rich model for chemoresistant mesenchymal-like basal breast cancers confers a significant benefit, largely through suppression of TAM motility. The orthotopic Py8119 mammary tumor model is renowned for its high expression of CSF-1, recruitment of large numbers of TAMs and aggressively invasive behavior (12, 16). In an allelic series of HCK mutant hosts, we demonstrate that increased HCK activity promotes Py8119 mammary tumor growth. Conversely, therapeutic inhibition of HCK reduced Py8119 tumor growth *in vivo*. Thus, HCK could provide a myeloid cell-specific therapeutic target to limit tumor progression through complementary cellular mechanisms mediated directly and indirectly by TAMs.

## Materials and methods

### Animal ethics

*In vivo* mammary tumor growth experiments were carried out strictly according to the ethics requirements of the University of Western Australia, which permitted 4 tumors per mouse and a maximum tumor size of 2 tumors each measuring 10x10mm (RA/3/100/1504, RA/3/100/1540). Hck-deficient (*Hck^KO^*), constitutively active Hck (*Hck^CA^*) and therapeutic RK20449 experiments were carried out at the Olivia Newton John Cancer Research Institute under an ethics approval permitting one tumor per mouse with a maximum tumor volume of 1000mm^3^ (Austin Health A2021-5746).

### Orthotopic mammary tumor models

All animal studies were approved and conducted in accordance with the Animal Ethics Committees for either the University of Western Australia (prophylactic inhibitor experiments) or the Olivia Newton John Cancer Research Institute (HCK mutant mice and therapeutic inhibitor experiments). The University of Western Australia ethics approval permitted injection of 1x10^6^ Py8119 cells (ATCC Cat<ns/> CRL-3278, RRD: CVCL_AQ09) into each of four mammary fat pads (<ns/>3 and <ns/>4 bilaterally) of female C57BL/6 mice (RRD: IMSR_JAX:000664) aged 10–12 weeks of age sourced from the Animal Resources Centre (WA). Inhibitors were started the same day as the tumor cell injections. For HCK inhibition, 30 mg/kg RK20449 (10 mice)(synthesized by Reagency) or vehicle (10 mice)(10% Captisol) was injected twice daily by intraperitoneal (IP) injection. For PI3K p110δ inhibition, mice were treated with 30 mg/kg GS-9820 (9 mice)(Gilead Sciences, Foster City, CA) and compared to the CSF-1R inhibitor GW-2580 (5 mice)(80 mg/kg, Calbiochem, San Diego, CA) or vehicle (9 mice)(0.5% w/v methylcellulose/0.1% Tween 80) twice daily by oral gavage.

For the Hck-deficient (*Hck^KO^*) {Lowell et al., 1994 <ns/>101442}, constitutively active Hck (*Hck^CA^*) {Ernst et al., 2002 <ns/>61021}, and therapeutic RK20449 studies, Olivia Newton John Cancer Research Institute ethics approval permitted one tumor per mouse with 1x10^6^ Py8119 cells injected into the right inguinal mammary fat pad (<ns/>4). For therapeutic RK20449 administration, either RK20449 (30 mg/kg) or Captisol were commenced when tumors became palpable (Day 8). For CD8^+^ T cell and NK cell depletion, female *Hck^WT^*, *Hck^KO^* and *Hck^CA^* mice were given three 200µg doses of either IgG (5 mice), αCD8 (RRID: AB_322770, JPP Biologics)(5 mice) or αNK1.1 (RRID: AB_630043, JPP Biologics)(5 mice/group) prior to tumor cell inoculation and then continued every three days until the experimental endpoint.

### Py8119 tumor processing

Tumors were fixed in 4% paraformaldehyde (PFA, EMS, Hatfield, PA) and paraffin embedded (FFPE) or snap frozen for subsequent RNA or protein extraction. For single cell analyses, tumors were minced with scalpel blades and dissociated in 0.1 mg/ml DNAse 1 (Merck, Bayswater, Vic) and 1.5 mg/ml collagenase type 4 (Worthington, Lakewood, NJ) for 1 hour at 37 °C with an additional 0.1 mg/ml of DNAse 1 added for 30 minutes then passed through a 100µM cell strainer followed by red cell lysis. Aliquots of 1x10^6^ cells were stored at -80 °C in 50% FCS, 10% DMSO in F12K media.

### Macrophage extraction and cell culture

Bone marrow was flushed from the femurs and tibiae of 8–10 week old C57Bl/6 mice. Non-adherent mononuclear phagocytic precursor cells were differentiated into mature BMM in increasing doses of CSF-1 as described in Murrey et al. (Murrey et al., 2020). BMM were cultured in 120 ng/ml CSF-1 (kind gift of Dr E.R. Stanley) in α+ MEM (Life Technologies, NY) containing 10% fetal calf serum (FCS, Bovogen, Melbourne, Vic) with 10, 000U/ml penicillin and streptomycin (Thermo Fisher Scientific, Scoresby, Vic). Py8119 cells were grown in Ham’s F-12K media (Thermo Fisher Scientific) with 5% FCS, 0.1% Mito serum extender (Corning, Clayton, Vic), 50 µg/ml gentamicin and 2.5 µg/ml amphotericin B (Thermo Fisher Scientific).

### Quantitative PCR

RNA extraction was carried out using the RNeasy protocol and converted to cDNA using Omniscript reverse transcriptase, according to the manufacturer’s instructions (Qiagen, Clayton, Vic). Mouse oligonucleotide primers were as previously described (10). PCR amplification was carried out using a Bio‐Rad iQ5 light cycler using iQ SYBR Green Supermix (Bio‐Rad, Gladesville, NSW). The mRNA expression of individual SFKs was determined relative to RPLPO and GAPDH and corrected for the efficiency of each PCR reaction.

### Inhibitor growth curves

Py8119 cells were seeded in 96 well plates at 5x10^3^ cells per well and four wells per condition. Cells were allowed to settle for one hour prior to treatment with media containing either 10nM RK20449 or 30nM Dasatinib or Captisol/DMSO as control then the assay plates were inserted into an IncuCyte Zoom incubator (RRID: SCR_019874, Essen Bioscience). Four phase-contrast images were taken per well every two hours for 48 hours. Incucyte software measured cell growth as percent confluence. Significance testing was carried out on the doubling times of each condition using a one-way ANOVA, N = 4.

### Immunohistochemistry

Dewaxed 5µm thick FFPE tumor sections were subjected to heat mediated antigen retrieval in citrate (pH6) buffer with 0.05% Tween 20. EDTA (pH9) buffer was used for phosphospecific antibodies. After antigen retrieval, sections were incubated with 3% H_2_O_2_ for 5 minutes to block endogenous peroxidases followed by permeabilization in 0.1% Triton-X 100 in TBS for 10 minutes. Tissue sections were then blocked with 5% BSA in TBS for 30 minutes then incubated with primary antibody diluted in 1% BSA TBS overnight at 4 °C. Prediluted EnVision peroxidase system (DAKO/Agilent, Musgrave, Vic) was used with DAB for visualisation (Vector Laboratories, Newark, California). Tissues were counterstained with Gills hematoxylin and mounted in DePeX (BDH Laboratory Supplies, Poole, UK). To compare TAMs in vehicle and drug-treated tumor margins, detect ionized Ca-binding adapter molecule 1 (IBA1)^+^ cells per area of tumor tissue within a 500µm circumference of the tumor edge were quantified using CellProfiler Image Analysis Software (RRID: SCR_007358). To distinguish tumor centers from the margins, the center was measured as the area 1mm from the tumor edge. Areas of necrosis or tissue artifact were eliminated from the analysis.

For multiplex immunofluorescent IHC, antigen retrieval was carried out as above followed by permeabilization with 0.1% Triton-X 100. Tissues were blocked with 5% BSA in TBS with DAPI (Thermo Fisher Scientific) for 30 minutes then incubated with pooled primary antibodies in 1% BSA in TBS for 1 hour, washed and incubated with pooled secondary antibodies for another hour. Tissue autofluorescence was quenched in 10mM CuSO_4_ and 50mM (NH_4_)_2_SO_4_ for 10 minutes (17) and mounted in Prolong Diamond (Thermo Fisher Scientific). Slides were imaged on a Nikon A1R confocal microscope with a CFI Plan Apochromat Lambda 20x objective, NA 0.75 using NIS-Elements software (RRID: SCR_014329).

### Antibodies

See **Supplementary Table 1** for a list of IHC antibodies.

### Quantification of pY SFK signal and T cell numbers

Quantification of phospho-SFK staining was carried out using Fiji image analysis software (RRID: SCR_002285) (18). Briefly, the auto-threshold function was used to generate a region of interest around Iba1^+^ cells and the mean gray signal of pY-SFK staining was recorded within this region. For quantification of (CD3/CD8/Perforin) positive cells, stained cells were counted using the multi-point tool. A minimum of four tumors were imaged per condition, and quantification was carried out on at least two fields per tumor.

### Flow cytometry

Dissociated tumor cells (2x 10^6^) were blocked in FACS buffer (PBS with 2% FCS) with FC block (1:1000, BD Biosciences) for 10 minutes on ice then washed with PBS and stained with yellow Live/Dead viability dye (L34959, Thermo Fisher Scientific) for 30 minutes in the dark. Cells were washed again before staining for surface markers for 30 minutes (CD3, BD Biosciences <ns/>741716; CD8, BD Biosciences <ns/>755241). After a final wash, flow cytometry was performed using a BD Fortessa LSR SOPR. Data were analysed using Flow Jo v10.

### 10x genomics chromium library construction, sequencing and analysis

The cryopreserved dissociated tumor cells were recovered according to the Thawing Dissociated Tumor cells for Single Cell RNA Sequencing Demonstrated Protocol (CG000233 Rev A, 10x Genomics). Single cell libraries from three vehicle and three RK20449-treated tumors were constructed according to the Chromium Next GEM Single Cell 3’ Reagent kits v2 with approximately 9000 cells captured per sample. Subsequent single cell libraries were constructed from early (3) and late (3) tumors, Hck^WT^ (3) and Hck^KO^ (3) tumors, and Hck^WT^ (2) and Hck^CA^ (2) tumors according to the Chromium Next GEM Single Cell 3’ Reagent kits v3.1 (Dual Index) User Guide (CG000315 Rev E) with approximately 6000 cells per sample. All 22 single cell libraries were sequenced on the Illumina NovaSeq 6000. The BCL sequencing files were demultiplexed and converted into FASTQ using the bcl2fastq utility of Illumina BaseSpace Sequence Hub (RRID: SCR_015058). FASTQ files were processed using Cell Ranger 7.0.0 (RRID: SCR_017344) using the Cell Ranger-compatible transcriptome *refdata-gex-mm10-2020-A* reference. Both intronic and exonic reads were counted. Low quality cells were filtered by removing cells that expressed either fewer than 200 genes or more than 10% mitochondrial genes. The data were then integrated using Seurat v.5 (RRID: SCR_016341) as described at the SCTransform workflow using Seurat toolkit (version 5) (https://satijalab.org/seurat/articles/integration_introduction) (19). A clustering resolution of 0.7 was used to produce 25 clusters. After the first integration, doublets expressing mixed genes were removed and the object was re-clustered. Clusters were annotated using marker genes generated by Seurat findallmarkers function and further informed by canonical marker genes based on the literature (20–22). Two-dimensional unified manifold approximation and projections (UMAP) were created using Seurat. Violin plots were created using the scCustomize package (RRID: SCR_024675) (23). CellChat was used for the ligand-receptor analysis (24) Gene set enrichment analysis was carried out using the Enrichr web tool (RRID: SCR_001575) (25). An FDR cutoff of 0.05 was used.

### Co-culture Matrigel invasion assays

Py8119 cells and mature BMM were used for the Py8119/BMM invasion assays. Either 4x10^4^ Py8119 cells, or 4x10^4^ Py8119 cells in the invasion chamber with 2x10^4^ BMM in the lower chamber or 4x10^4^ Py8119 cells + 2x10^4^ BMM were placed together in 500µl CSF-1-containing media in BD Biocoat 8 µM pore sized 24 well-well Matrigel-coated inserts (BD Biosciences). The inserts were then placed in wells containing 500µl media for 18 hours. Invasive Py8119 cells were fixed in 4% PFA then counted (10 fields/insert). To correct for cell loading, 2x10^4^ Py8119 cells were added to insert-free wells. For AXL inhibitor experiments, either DMSO or R428 (0.5µM) was added to the insert along with the cells.

### Statistical analysis

GraphPad Prism 9.5 (RRID: SCR_002798) was used to carry out statistical analysis for all *in vitro* assays and for analysis of tumor weights from animal experiments. Values are displayed as mean +/- SEM or SD. Students T-test (two-tailed, unpaired) was used to compare the means of two groups. For three or more groups, one-way ANOVA was used with Tukeys *post-hoc* correction. P values <0.05 were considered significant. Seurat findmarkers was used to calculate differentially expressed genes. Adjusted P values <0.05 were considered significant and differentially expressed genes expressed in less than 5% of either condition or with a LFC of less than 0.25 were excluded.

## Results

### Inhibition of macrophage motility reduces Py8119 mammary tumor growth

**Figure 1:**
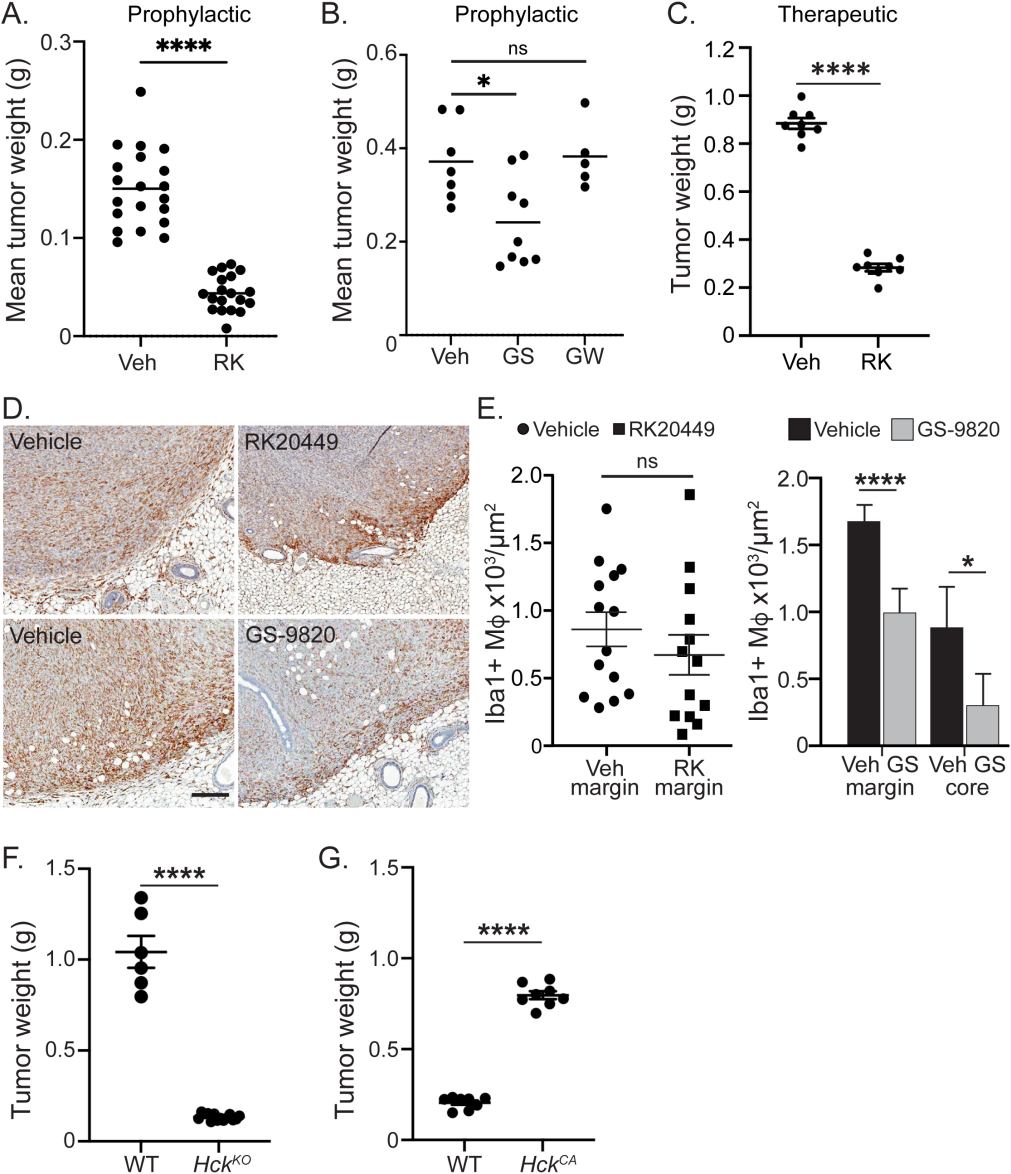
HCK and PI3K p110δ regulate Py8119 mammary tumor growth. **(A)** Tumor weights of mice treated prophylactically with the HCK inhibitor RK20449 (RK). Data points represent the mean tumor weight of four individual tumors inoculated/mouse and data are represented as mean ± SEM and significance testing was conducted using student’s T-test. N = 10 vehicle and 10 RK20449-treated mice. **(B)** Tumor weights of mice treated prophylactically with the PI3K p110δ inhibitor GS9820. Data points represent the mean tumor weight of four individual tumors inoculated/mouse and data are represented as mean ± SEM. Significance testing was conducted using one way ANOVA. N = 9 vehicle, 9 GS-9820- and 5 GW2580-treated mice. **(C)** Tumor weights of mice treated therapeutically with the HCK inhibitor RK20449 (RK). Data points represent individual tumor weights with one tumor inoculated/mouse and data are represented as mean ± SEM and significance testing was conducted using student’s T-test. N = 8 vehicle and 8 RK20449-treated mice. **(D)** IBA1^+^ TAMs in tumor sections from vehicle vs RK20449-treated mice and vehicle vs GS-9820-treated mice. Scale bar = 200µm. **(E)** Quantification of TAMs at the tumor margins in vehicle and RK20449-treated tumors and at the tumor margins and cores in vehicle and GS9820-treated tumors. Data are represented as mean ± SEM and significance testing was conducted using student’s T-test. N = 14 vehicle and 12 RK20449-treated tumors. **(F)** Tumor weights of *Hck^WT^* and *Hck^KO^* mice. Data points represent individual tumor weights with one tumor inoculated/mouse and data are represented as mean ± SEM and significance testing was conducted using student’s T-test. N = 6 *Hck^WT^* and 14 *Hck^KO^* mice. **(G)** Tumor weights of *Hck^WT^* and *Hck^CA^* mice. Data points represent individual tumor weights with one tumor inoculated/mouse and data are represented as mean ± SEM and significance testing was conducted using student’s T-test. N = 8 *Hck^WT^* and 8 *Hck^CA^* mice. For all statistical comparisons, ns denotes not significant, *p < 0.05, ****p < 0.0001.

To determine whether HCK activity regulates macrophage motility and associated invasive growth, we took advantage of the fact that HCK becomes activated in response to ligand engagement of the CSF-1R with subsequent stimulation of macrophage motility. Accordingly, we inhibited HCK activity with the pyrrolo-pyrimidine compound RK20449 (26), which we have previously shown to block motility and invasive capacity of both WT and *Hck^CA^* BMM *in vitro* (10, 13), and to reduce tumor growth in colorectal, gastric and pancreatic cancer *in vivo* (13–15). Female mice were injected with Py8119 cells and RK20449 injections started immediately, thereby reducing mammary tumor growth by 70% (3.3-fold) (**Figure 1A**). We then observed that inhibition of PI3K p110δ with the selective inhibitor GS-9820 (acalisib) reduced tumor size by 35% (1.5-fold) (**Figure 1B**) (27). In contrast and consistent with previous reports in PyMT mice, pan-CSF-1R inhibition by GW-2580 did not reduce Py8119 tumor growth (**Figure 1B**) (28).

The therapeutic efficacy of HCK inhibition was tested by administering RK20449 when tumors became palpable. Tumor growth was again reduced by 70% (**Figure 1C**). To exclude the possibility that RK20449 treatment would directly affect the growth of Py8119 cells, which do not express HCK (**Supplementary Figures 1A, B**), we determined their doubling time and found that it was unaffected by RK20449 but suppressed by dasatinib, a pan-SFK inhibitor (**Supplementary Figures 1C, D**).

To determine whether motility inhibition affected TAM numbers or distribution in Py8119 tumors, immunohistochemistry (IHC) was used with IBA1 as a macrophage marker. Abundant TAMs were seen in control tumors, particularly at the tumor edge, and these were reduced in number by PI3K p110δ inhibition (**Figures 1D, E**). In contrast, HCK inhibition did not impact TAM numbers or their accumulation at the tumor edge (**Figures 1D, E**). Taken together, inhibition of CSF-1R-activated macrophage motility but not full CSF-1R blockade reduced Py8119 mammary tumor growth with HCK inhibition being twice as effective as PI3K p110δ inhibition. Moreover, RK20449 inhibited tumor growth without any detectable effects on TAM number or distribution.

### HCK activity correlates with Py8119 mammary tumor growth

Because HCK inhibition profoundly reduced Py8119 tumor growth and hyper-motile *Hck^CA^* BMM promoted increased tumor cell invasion in BMM-infiltrated Py8119 mammospheres (13), tumor growth was examined in hosts either deficient in HCK expression (*Hck^KO^*) or expressing constitutively active HCK (*Hck^CA^*). *HCK*-deficient hosts limited growth by more than 7-fold compared to WT hosts (WT mean 1.04g, *Hck^KO^* mean 0.134g) whereas tumors grew to 4-fold increased size in hosts expressing the HCK^CA^ isoform relative to tumor size in WT hosts (WT mean 0.206g, *Hck^CA^* mean 0.797g) (**Figures 1F, G**). Although Py8119 mammary tumor ulceration into overlying skin is typically very infrequent (LG Ellies, unpublished observations), ulceration occurred in four of eight *Hck^CA^* mice, triggering early euthanasia and smaller tumor size for the cohort.

### Loss of HCK activity reduces SFK activity in Py8119 tumor margins

**Figure 2:**
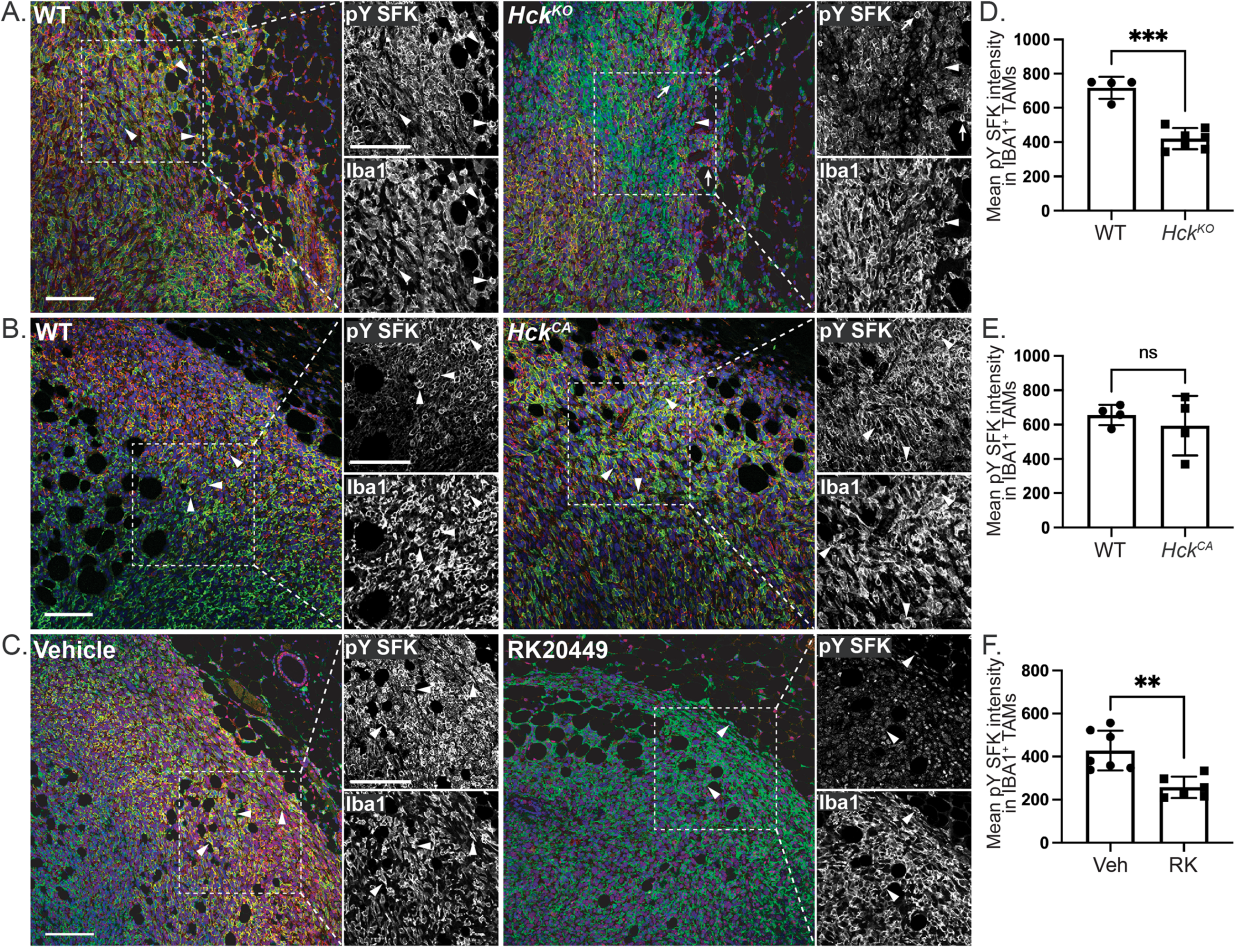
Hck regulates membrane-associated SFK activity in Py8119 tumors. **(A)** *Hck^WT^* and *Hck^KO^* tumor sections stained for IBA1 (green), pY416 SFK (red) and DAPI (blue). Dashed insets show outlined regions at higher magnification. **(B)** *Hck^WT^* and *Hck^CA^* and **(C)** vehicle and RK20449-treated tumor sections similarly stained. Quantification of mean pY SFK signal intensity for IBA1^+^ TAMs in *Hck^WT^*, *Hck^KO^* **(D)**, *Hck^WT^*, *Hck^CA^* **(E)** and vehicle, RK20449-treated **(F)** Py8119 tumor margins. Scale bars, 200µm. Mean ± SEM, ns, not significant, **p < 0.01, ***p < 0.001.

Because HCK activity correlated positively with tumor growth, we used a pY410-specific HCK antibody to examine the distribution of HCK activity by IHC. However, strong nuclear staining in IBA1^+^ TAMs and other cells in *Hck^KO^* tumors indicated a lack of specificity of the pY-HCK antibody for HCK (**Supplementary Figure 2**). In contrast, a pY416-SFK antibody that detects all activated SFKs, including HCK, demonstrated plasma membrane-associated SFK activity at the margins of IBA1^+^ TAMs and other cells in Py8119 tumors in treatment-naïve and vehicle-treated *Hck^WT^* hosts (**Figures 2A-C**, arrowheads). This membrane-associated SFK activity was absent at the margins of tumors recovered from *Hck^KO^* and RK20449-treated *Hck^WT^* hosts (**Figures 2A, C**, arrowheads). IBA1^-^ pYSFK^+^ cells in *Hck^KO^* tumors likely represent T cells (**Figure 2A**, arrows). As RK20449 also inhibits LCK, the primary SFK in T cells, IBA1^-^ pYSFK^+^ cells were not seen in RK20449-treated *Hck^WT^* tumors (**Figure 2C**). Quantification of pY SFK intensity in IBA^+^ cells confirmed the reduction in signal for TAMs in *Hck^KO^* and RK20449-treated *Hck^WT^* tumors (**Figures 2D, F**). In contrast, pY SFK signal intensity in TAMs was not measurably increased in *Hck^CA^* tumors (**Figures 2B, E**). Together, these results indicate that membrane-associated SFK activity is seen in IBA1^+^ TAMs at the invasive margins of Py8119 mammary tumors, provided HCK is both expressed and active.

### HCK regulates cytotoxic T cell numbers in Py8119 tumors

**Figure 3:**
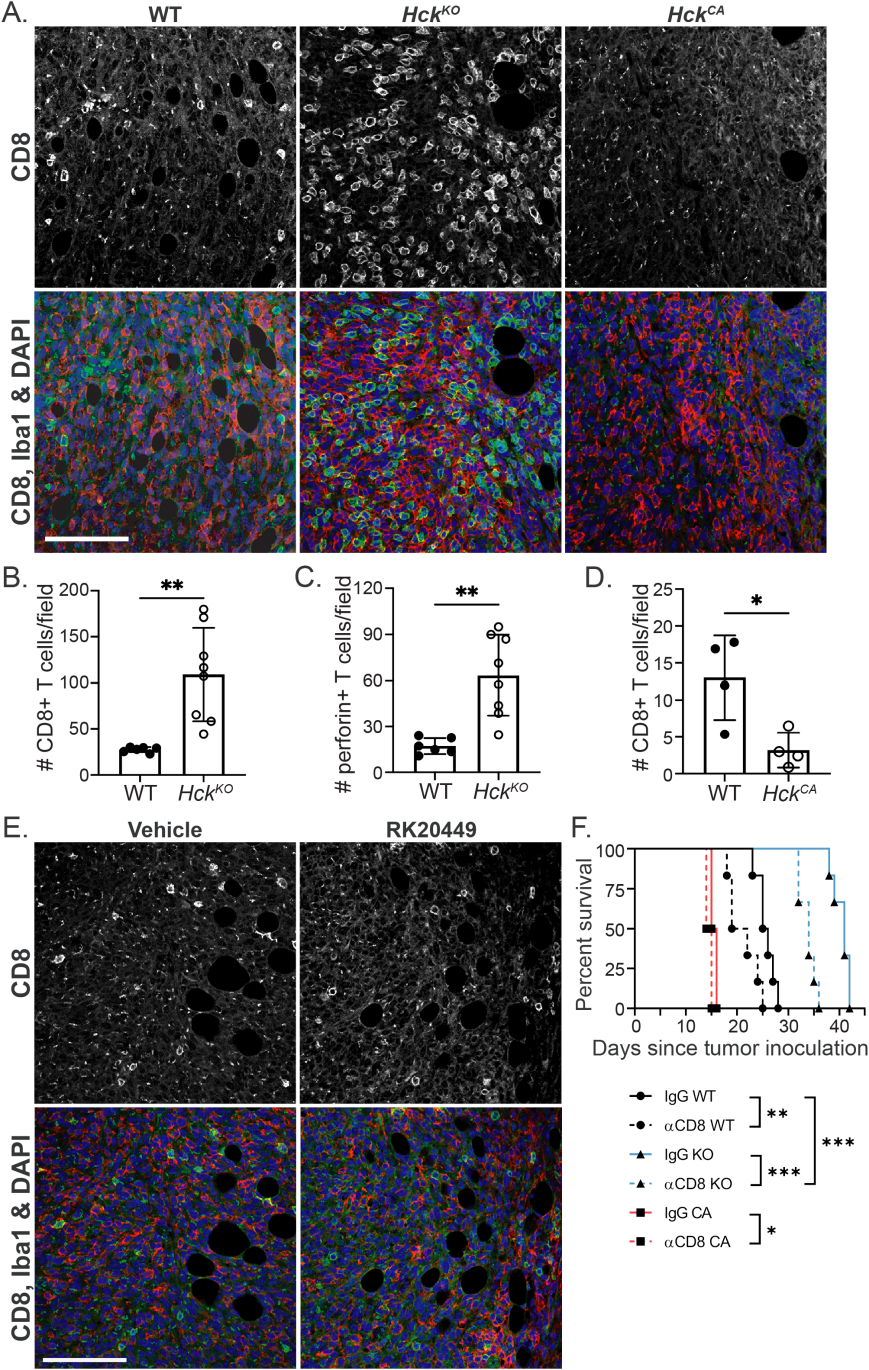
HCK activity regulates CD8^+^ T cell numbers in Py8119 tumors. **(A)** *Hck^WT^*, *Hck^KO^* and *Hck^CA^* tumor sections stained for CD8 (green), IBA1 (red) and DAPI (blue). **(B, C)** Quantification of CD8^+^ and perforin^+^ T cells in *Hck^WT^* and *Hck^KO^* tumors respectively and, **(D)** CD8^+^ T cells in *Hck^WT^* and *Hck^CA^* tumors. **(E)** Vehicle and RK20449-treated tumor sections stained for CD8 (green), IBA1 (red) and DAPI (blue). **(F)** Survival of Py8119 tumor-bearing *Hck^WT^* (circles, black lines), *Hck^KO^* (triangles, blue lines) and *Hck^CA^* (squares, red lines) mice treated with anti-CD8 antibody (solid lines) or IgG control (dashed lines). Scale bars, 200µm. Mean ± SEM, *p < 0.05, **p < 0.01, ***p < 0.001.

We have shown in other tumor models that HCK deletion or inhibition increases cytotoxic T cell recruitment and activation (8, 15). This may account for some of the effect on Py8119 tumor growth conferred by the level of HCK expression in the host. Consistent with this, both CD8^+^ T cell abundance and activity (perforin) were increased by 4-fold in tumors from *Hck^KO^* hosts while scant CD8^+^ T cells were seen in tumors from *Hck^CA^* hosts (**Figures 3A-D**). Flow cytometry confirmed these findings with a 4-fold increase in CD3^+^ T cell numbers and a 3.5-fold increase in CD8^+^ T cells in *Hck^KO^* tumors (**Supplementary Figure 3A**). Overall, CD3^+^ T cells comprised approximately 3% and 10% of cells in *Hck^WT^* and *Hck^KO^* tumors respectively. In contrast, flow cytometry of *Hck^WT^* and *Hck^CA^* tumors did not show any apparent differences in CD3^+^ and CD8^+^ T cell numbers (**Supplementary Figure 3B**). RK20449 did not change CD8^+^ T cell numbers (**Figure 3E**), perhaps due to its known affinity for the T cell-specific SFK member LCK. Thus, HCK activity in TAMs appears to regulate cytotoxic T cell numbers and activity in Py8119 tumors.

An anti-CD8^+^T cell-depleting antibody (YTS169) was used to quantitatively examine the effect of CD8^+^ T cells on Py8119 tumor growth. *Hck^WT^*, *Hck^KO^* and *Hck^CA^* hosts were injected with the antibody prior to, during and after tumor cell inoculation. CD8^+^ T cell depletion reduced survival of tumor-bearing *Hck^WT^* hosts from 25.5 to 20.5 days (p<0.01), from 41.0 to 34.0 days (p<0.001) of *Hck^KO^* hosts, and from 15.5 to 14.5 days (p<0.05) of *Hck^CA^* hosts (**Figure 3F**). However, following T cell depletion, *Hck^KO^* hosts survived 14 days longer than *Hck^WT^* hosts lacking cytotoxic T cells (p<0.0001) and 8.5 days longer than *Hck^WT^* mice treated with an isotype IgG control (p<0.001). NK cell depletion had no effect on tumor growth in *Hck^WT^* or *Hck^CA^* hosts and only a small effect on *Hck^KO^* hosts (**Supplementary Figure 3C**). These results demonstrate that the effect of HCK inhibition is only partly mediated by adaptive immune responses, which is consistent with our findings in MC38 colon and KPC pancreatic tumors (8, 15).

### TAMs comprise more than 40% of Py8119 mammary tumor mass

**Figure 4:**
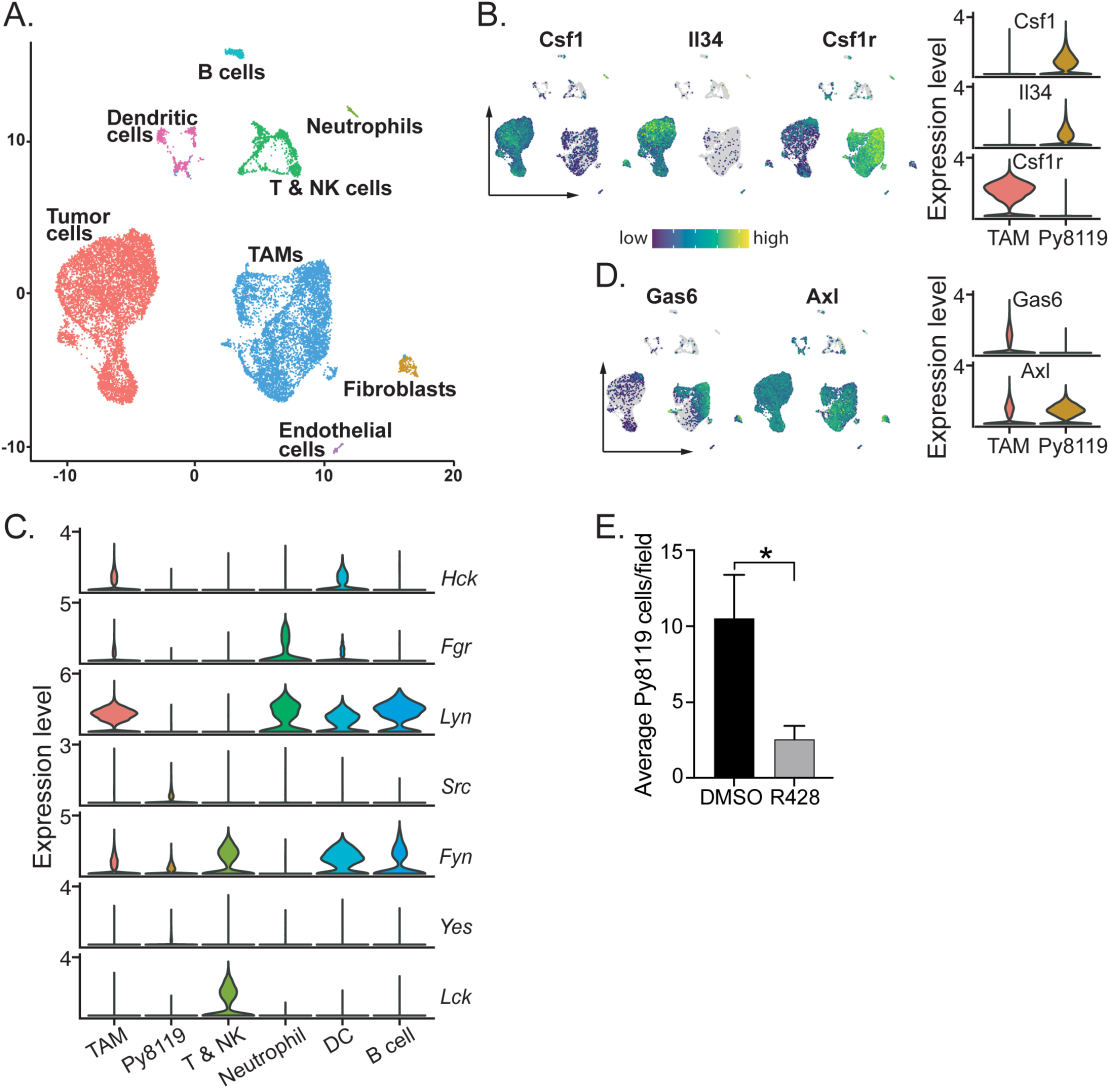
Py8119 cells and TAMs set up a CSF-1, IL-34/GAS6 paracrine loop to recruit abundant TAMs and activate Py8119 cell invasion. **(A)** Uniform manifold approximation and projection (UMAP) plot and graph-based clustering of early (day 10) and late (day 14) *Hck^WT^* tumors. **(B)** UMAP and violin plots depicting expression of CSF-1, IL-34 and CSF-1R in tumor cells and TAMs. **(C)** Violin plots depicting SFK gene expression in Py8119 tumors. **(D)** UMAP and violin plots showing expression of GAS6 and AXL in TAMs and tumor cells. **(E)** Py8119 cell Matrigel invasion *in vitro* with co-cultured BMM in the presence of DMSO or R428 (0.5µM). Data are represented as mean ± SEM and significance testing was conducted using student’s T-test. *p < 0.05.

To further examine the role of HCK in Py8119 tumors, single cell RNA sequencing (scRNA-seq) was carried out on tumors harvested from RK20449-treated, *Hck^KO^*, *Hck^CA^* and matched control hosts. Because the rapid growth and high ulceration rate of tumors in *Hck^CA^* hosts necessitated the recovery of smaller tumors from matched WT hosts (0.206g) than typical end-stage tumors recovered from WT hosts when paired with *Hck^KO^* hosts (1.04g), initial analyses compared 10 day (early) and 14 day (late) WT tumors to determine whether tumor size affected its cellular composition. Transcriptomes of 16, 477 cells were sequenced, filtered and normalized to cluster cells in an unbiased manner with annotation based on differentially expressed genes (DEGs) in Seurat and on lineage specific markers (**Supplementary File 1**). TAMs comprised 42.6% of cells in early tumors and 42.1% in end-stage tumors with 43.5% and 39.1% tumor cells respectively (**Table 1**; **Figure 4A**).

**Table 1:** Cell numbers.

Cell Type	Total N° (%)	Vehicle N° (%)	RK N° (%)	Hck^WT^ N° (%)	Hck^KO^ N° (%)	Hck^WT^ N° (%)	Hck^CA^ N° (%)	Mid N° (%)	Late N° (%)
TAMs	45472 (44.0)	12351 (47.5)	13090 (45.9)	3433 (40.6)	3463 (41.4)	4406 (44.1)	2020 (34.7)	4000 (42.5)	2979 (42.1)
Py8119 cells	45864 (44.4)	10566 (40.7)	12697 (44.5)	4078 (48.2)	3607 (43.2)	4886 (48.9)	3146 (54.0)	4086 (43.5)	2798 (39.5)
T & NK cells	5658 (5.5)	1743 (6.7)	830 (2.9)	387 (4.6)	888 (10.6)	299 (3.0)	337 (5.8)	544 (5.8)	621 (8.8)
Dendritic cells	2306 (2.2)	769 (3.0)	485 (1.7)	119 (1.4)	139 (1.7)	177 (1.8)	69 (1.2)	265 (2.8)	283 (4.0)
Neutrophils	1680 (1.6)	275 (1.1)	840 (2.9)	193 (2.3)	80 (1.0)	42 (0.4)	27 (0.5)	121 (1.3)	102 (1.4)
B cells	1169 (1.1)	81 (0.3)	233 (0.8)	155 (1.8)	131 (1.6)	90 (0.9)	173 (3.0)	190 (2.0)	116 (1.6)
Fibroblasts	647 (0.6)	107 (0.4)	193 (0.7)	27 (0.3)	19 (0.2)	33 (0.3)	21 (0.4)	125 (1.3)	122 (1.7)
Endothelial cells	531 (0.5)	84 (0.3)	129 (0.5)	65 (0.8)	29 (0.4)	66 (0.7)	33 (0.6)	70 (0.7)	55 (0.8)
Total	103327 (100)	25976 (100)	28506 (100)	8457 (100)	8356 (100)	9999 (100)	5826 (100)	9401 (100)	7076 (100)

As tumor stage did not affect Py8119 tumor composition, tumors from all genotypes, treatment groups and stages were then combined (>103, 000 cells), clustered and annotated. Overall, TAMs made up 44.0% of cells compared to 44.4% tumor cells, 5.5% T and NK cells with small clusters of other cell types (**Table 1**). High expression by Py8119 cells of two macrophage chemokines, CSF-1 and interleukin (IL)-34, likely underpinned the recruitment of so many CSF-1R^+^ TAMs (**Figure 4B**). Consistent with the IHC findings, separation of the scRNA-seq data by HCK treatment or genotype demonstrated that TAM numbers were not reduced by loss of HCK activity (**Table 1**). In contrast, TAM numbers appeared to be reduced in *Hck^CA^* tumors (**Table 1**). Also consistent with the IHC and flow cytometry findings, T and NK cell numbers were doubled by *Hck* deletion (4.6 to 10.6%, p = 0.039) while RK290449 treatment reduced them (6.7 to 2.9%), the latter finding probably reflecting off-target LCK inhibition in T cells. Finally, scRNA-seq data from tumors of *Hck*-replete hosts confirmed that TAMs and DCs expressed HCK and other myeloid SFKs whereas T and NK cells expressed LCK and FYN and Py8119 cells expressed low levels of the ubiquitous SFKs only (**Figure 4C**).

### Py8119 cells and TAMs form a CSF-1/Gas6 paracrine loop to drive Py8119 cell invasion

Tumor invasion in spontaneous PyMT tumors is driven by a CSF-1/EGF paracrine chemotactic loop (29). As mentioned above, Py8119 cells expressed high levels of both CSF-1 and IL-34 (**Figure 4B**). However, although Py8119 cells express the EGFR, TAMs did not express EGF or other high affinity EGFR ligands such as TGFα and betacellulin (**Supplementary Figure 4A**). To identify alternative Py8119 cell chemokines secreted by TAMs, potential ligand-receptor interactions between TAMs and tumor cells were analysed using the cell-cell communication analysis tool CellChat (29). The strongest TAM chemokine ligand/Py8119 cell receptor combination was growth arrest specific 6 (Gas6) and AXL with a large subset of TAMs expressing the chemokine while tumor cells and TAMs expressed the AXL receptor tyrosine kinase (**Figure 4D**; **Supplementary Figures 4B, C**). To confirm that AXL played a role in Py8119 cell invasive capacity, a Matrigel *in vitro* invasion assay was used in which Py8119 cells are only invasive when in direct contact with co-cultured BMM (**Supplementary Figure 4D**). A selective AXL inhibitor R428 (0.5µM) reduced Py8119 cell invasion into Matrigel 4-fold (**Figure 4E**) (30). Thus, CSF-1 and Gas6 form a paracrine chemokine loop to drive co-invasion of Py8119 cells and TAMs.

### The five TAM subtypes in Py8119 tumors are all pro-tumoral

**Figure 5:**
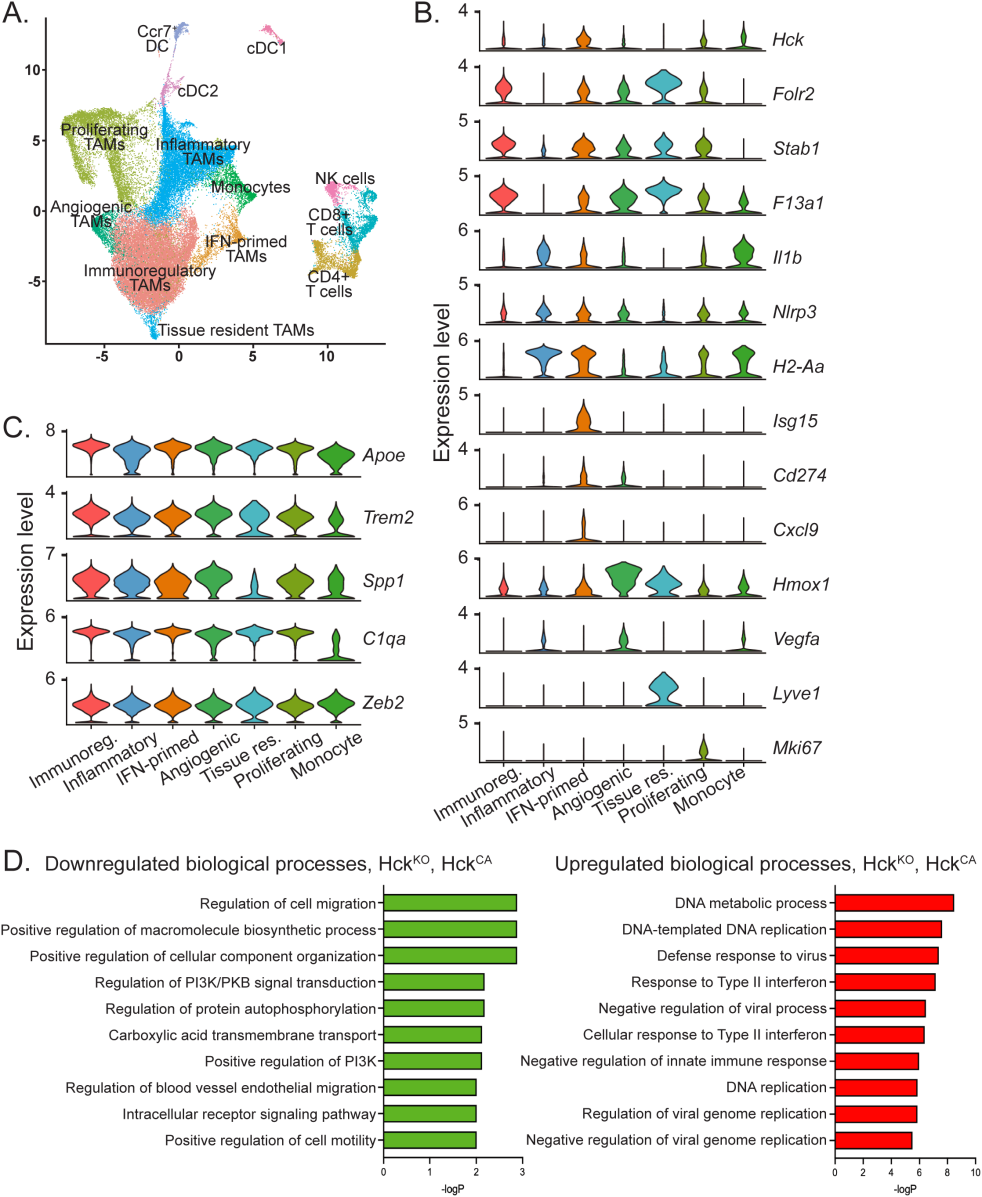
TAMs in Py8119 tumors are comprised of 5 pro-tumoral sub-types and HCK deficiency does not alter sub-type proportions but downregulates TAM migration. **(A)** UMAP plot of CD45^+^ cells from *Hck^WT^*, *Hck^KO^*, *Hck^CA^*, vehicle and RK20449-treated tumors. **(B)** Violin plots depicting expression of TAM sub-type cluster markers in TAM sub-types, proliferating TAMs and monocytes. **(C)** Violin plots depicting expression of M2-like pro-tumoral and M1-like anti-tumoral markers in TAM sub-types, proliferating TAMs and monocytes. **(D)** Pathway analysis of DEGs in *Hck^KO^* and *Hck^CA^* TAMs using the Enrichr biological processes gene set library.

To examine the role of HCK in the differentiation of TAM phenotypes, CD45^+^ immune cells were reclustered (**Figure 5A**). We and others have devised a consensus classification of macrophage subtypes based on single cell data across a range of tumor types to distinguish between angiogenic, immunoregulatory, interferon (IFN)-primed, inflammatory, lipid-associated, tissue resident and proliferating TAMs (3, 4). TAMs in Py8119 tumors consisted of a large immunoregulatory cluster (>40%, folate receptor beta (Folr2)^high^, stabilin (Stab)1^high^, F13a1^high^), an inflammatory cluster (~20%, H2-Aa^high^, NLRP3^high^, Il1b^high^) and smaller clusters of IFN-primed (~5%, Isg15 ubiquitin like modifier (Isg15)^high^, Cd274^high^, Cxcl9), angiogenic (~4%, heme oxygenase (Hmox) 1^high^, Vegfa^high^), and tissue resident (~1%, lymphatic vessel endothelial hyaluronan receptor (Lyve)-1^high^) macrophages, with proliferating TAMs (Mki67^+^) accounting for 20% (**Figures 5A, B**). All TAMs extracted from Py8119 tumors expressed abundant apolipoprotein E (ApoE)^high^, reflecting their mammary fat pad origin, as well as triggering receptor expressed on myeloid cells (TREM2)^+^, secreted phosphoprotein 1 (Spp1)^high^), and C1q (**Figure 5C**). Similarly, HCK was expressed across all TAM subtypes (**Figure 5B**). Importantly, loss of HCK activity did not induce an anti-tumoral phenotype in any TAM clusters as determined by lack of expression of inducible nitric oxide synthase (Nos2)^+^ and other classically activated macrophage markers (**Supplementary Figure 5A**). Likewise, there was no induction of IL-10, matrix metalloproteinase (Mmp)2 or Mmp9 expression or changes in arginase (Arg)1 expression to indicate a skewing of TAMs towards an alternatively activated phenotype, as we have shown occurs in several gastrointestinal tumor models (**Supplementary Figure 5B**) (13–15).

### Gene set enrichment analysis demonstrates downregulation of cell migration in HCK-deficient TAMs

Since loss of HCK activity did not change the overall phenotype of TAMs in PY8*119* tumors, pathway enrichment analysis was carried out on TAMs from *Hck^KO^* and *Hck^CA^* tumors to identify biological processes significantly altered by HCK inactivation. DEGs of *Hck^KO^* and *Hck^CA^* TAMs were compared using Enrichr and the biological process gene set library (24). Consistent with a role for TAMs in the promotion of PY8119 tumor invasion, regulation of cell migration/motility were two of the most strongly downregulated biological processes in HCK-deficient TAMs whereas the majority of upregulated processes involved responses to interferons and viral infections (**Figure 5D**). Thus, HCK-activated phosphotyrosine-based motility signaling downstream of the CSF-1R in TAMs is a significant driver of PY8119 tumor growth. RK20449-treated TAMs revealed downregulation of cytokine pathways, and upregulation of metabolic processes whereas interferon signaling pathways were not increased, likely due to the lack of increase in T cells (**Supplementary Figure 5C**).

### Gene set enrichment analysis reveals downregulation of epithelial-to-mesenchymal transition but not proliferation in Py8119 tumor cells

**Figure 6:**
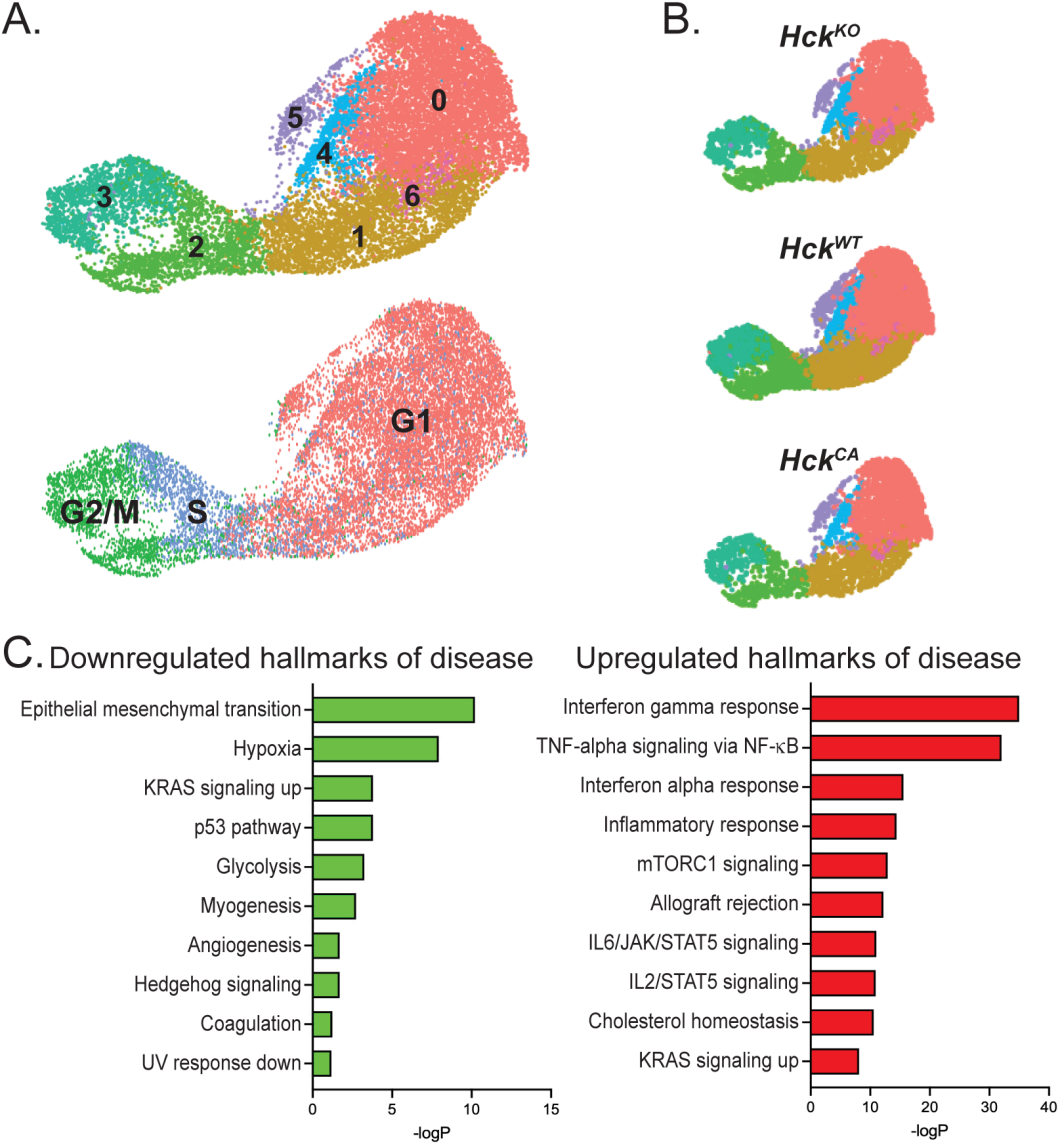
HCK activity in TAMs does not affect Py8119 tumor cell proliferation but reduces epithelial-to-mesenchymal transition **(A)** Collective UMAP plot of Py8119 tumor cells from *Hck^WT^*, *Hck^KO^* and *Hck^CA^* tumors. Proliferating Mki67^hi^ cells are shown in clusters 2 and 3. **(B)** Individual UMAP plots of Py8119 tumor cells from *Hck^WT^*, *Hck^KO^* and *Hck^CA^* tumors. **(C)** Pathway analysis of DEGs in tumor cells from *Hck^KO^* and *Hck^CA^* tumors using the Enrichr hallmarks of disease gene set library.

Py8119 cells from *Hck^WT^*, *Hck^KO^* and *Hck^CA^* host tumors were examined for evidence that HCK activity in TAMs also affected tumor cells. N-Cadherin^hi^, Twist1^hi^ and Col4a5^hi^ (collagen IV) cells were reclustered and formed seven closely associated clusters (**Figure 6A**). Highly proliferative (Mki67^hi^) cells were concentrated in clusters 2 and 3 and were shown by DEG analysis to be in S and G2/M phase (**Figure 6A**). Mki67^hi^ cells constituted 22.8% of tumor cells overall with similar proportions in Hck^WT^ (23%), Hck^KO^ (23%) and Hck^CA^ (21%) tumors (**Figure 6B**). These results indicate that HCK activity in TAMs does not influence tumor proliferation. Enrichr analysis using the hallmarks of disease gene set was then carried out to determine whether TAM HCK activity affected PY8119 tumor cells in other ways. Interestingly, epithelial-to-mesenchymal transition (EMT) was downregulated in *Hck^KO^* tumors as was hypoxia signaling whereas interferon and inflammatory response signals were upregulated (**Figure 6C**). Thus, HCK activity in TAMs indirectly reduced EMT at a transcriptional level in tumor cells, which provides further evidence for the role of TAM motility in enabling tumor cells to become invasive.

Overall, scRNA-seq revealed that TAMs comprised almost half the Py8119 tumor mass and differentiated into 5 pro-tumoral subtypes that were unaffected by HCK activity. Pathway analysis demonstrated that reduced TAM migration likely contributed to the reduction in invasive mammary tumor growth seen in *Hck^KO^* hosts compared to *Hck^CA^* hosts with additional evidence of an indirect effect on EMT in tumor cells.

## Discussion

TAMs are found in many solid tumors and their abundance correlates with poor survival (22, 31). A wealth of clinical and preclinical data has shown that most TAMs are monocyte-derived and recruited to tumors where they promote growth and dissemination through a variety of mechanisms (2). In breast and several other cancers, tumor cells have been shown to secrete CSF-1 to recruit TAMs, which often accumulate in invasive regions (32, 33). Consistent with this observation, primary breast cancers expressing high levels of CSF-1 are more likely to progress to metastasis and cause death (34). The critical role of CSF-1 for invasion and metastasis was highlighted by the observation that CSF-1-deficient PyMT mammary tumors rarely metastasized (35). A subsequent study demonstrated the existence of a paracrine chemokine loop between CSF-1-secreting tumor cells and EGF-secreting TAMs that underpinned PyMT tumor invasion and metastasis (29). However, despite the evidence for CSF-1 in the promotion of invasion and metastasis, clinical trials of CSF-1/CSF-1R inhibitors in several cancer types have shown limited efficacy, with the notable exception of tenosynovial giant cell tumors driven by a CSF1 rearrangement (36). Likewise, pan-CSF-1R inhibition also failed to reduce mammary tumor growth in PyMT mice or in our Py8119 model (**Figure 1B**) (27).

Hence, attention has shifted to targeting tumor promoting behaviors of TAMs with a focus on changing characteristics of TAMs from a pro-tumoral to an anti-tumoral phenotype (1, 2). As the absence of CSF-1 had a significantly greater effect on metastasis than progression of primary PyMT tumors, we examined whether targeting CSF-1-activated motility and invasive behavior of TAMs could reduce tumor growth and invasion in a PyMT-derived orthotopic mammary tumor model (16). These highly invasive Py8119 tumors secrete CSF-1 and IL-34 and recruit TAMs to ultimately contribute to over 40% of the total tumor mass. Here we show that targeting CSF-1-activated TAM invasion significantly reduces tumor growth and invasion (16).

We have previously shown that Py8119 tumor cells must be in direct contact with macrophages to become invasive and that contact with hyper-motile *Hck^CA^* macrophages doubles Py8119 cell invasion *in vitro* (10, 13). Similar mechanisms are likely to occur *in vivo* as TAMs containing active HCK accumulate at the invasive front of Py8119 tumors and, when TAM motility is selectively blocked by either HCK or PI3K p110δ inhibition, Py8119 tumor growth is reduced. In contrast, increased tumor growth with frequent ulceration is seen in hosts expressing constitutively active HCK, which is consistent with the increased invasiveness of gastric tumors in HCK^CA^ hosts (Poh et al., 2020). The combined observations of profoundly reduced Py8119 tumor growth in HCK-deficient hosts and rapid, invasive growth in HCK^CA^ hosts indicate that HCK activity in TAMs regulates tumor growth and invasion.

TAM promotion of invasive growth is consistent with the notion that TAMs recapitulate the behavior of macrophages during tissue development (37). CSF-1-dependent macrophages remodel the ECM to facilitate terminal end bud outgrowth during mammary ductal morphogenesis (5). In a similar manner, TAMs express an array of MMPs and cathepsins to digest collagen and other ECM proteins and facilitate tumor invasion (11). Moreover, constitutively active HCK in TAMs has been shown to cause increased tumor cell invasion in a gastric model of cancer (13). Combined with our observations that high levels of SFK activity are seen in TAMs located at tumor margins of Py8119 tumors in HCK-proficient but not HCK-deficient hosts, and the downregulation of cell migration pathways in TAMs in *Hck^KO^* tumors, it is likely that the HCK-dependent invasive activity of TAMs contributes to invasion of Py8119 cells (12, 38). Consistent with this, membrane-associated SFK activity is associated with reduced survival in triple negative breast cancer (TNBC) (39, 40). Taken together, our results indicate that CSF-1 signaling-dependent HCK activity and associated TAM motility promote invasive growth of these rapidly growing tumors.

Our finding that CD8^+^ T cell numbers are increased in tumors from *Hck^KO^* hosts indicates that myeloid HCK activity regulates CD8^+^ T cell infiltration into Py8119 tumors (8). The mechanism by which TAMs reduce cytotoxic T cell recruitment is unclear but macrophages have been shown to interact with CD8^+^ T cells in normal and cancerous breast tissue (41). Interestingly, mammary ductal macrophages extend highly dynamic branches to routinely survey the ductal epithelium (42). Analogously, TAMs in the tumor margins may extend branches to directly interact with T cells and regulate their behavior. As the pathways controlling these actin-rich extensions are the same as those regulating HCK-dependent macrophage motility, HCK deficiency could limit the dynamic behavior of these branches and increase CD8^+^ T cell recruitment. Consistent with this, we have shown that a combination of HCK and immune checkpoint inhibitors controls tumor growth across a range of otherwise immune cell excluded (“cold”) tumors (8, 15). Nevertheless, Py8119 tumors in CD8^+^ T cell-depleted HCK-deficient hosts grow even more slowly than tumors in CD8^+^ T cell-depleted, HCK-proficient hosts. Thus, we speculate that the extent of the contribution of HCK activity to limit T cell-dependent immune control and to promote invasion of tumor cells into the surrounding matrix may differ according to the oncogenic program specific to a particular malignancy or the target organ in which it occurs.

Similarly, the paracrine interaction driving co-invasion of Py8119 tumor cells and TAMs differs from spontaneous PyMT tumors. Gas6, which acts through the receptor tyrosine kinase AXL, has been associated with a poor prognosis and therapeutic resistance in solid cancers, including TNBC (43, 44). Thus, tumor cell chemokines driving invasion can vary, which may be a mechanism for treatment resistance. In contrast, CSF-1-driven TAM motility is common to both tumor models, underscoring the value of targeting HCK in genetically normal host cells to reduce tumor invasive growth.

A limitation of our study is that the PyMT tumor-derived Py8119 cell model is orthotopic whereas spontaneous PyMT tumors arise from the mammary ductal epithelium (16, 42, 45). Because TAMs differentiate in response to their local microenvironment, stromal and ductal TAM populations can be discerned in spontaneous but not orthotopic tumors (45). However, the majority of TAMs in spontaneous tumors are of the immunoregulatory and inflammatory phenotype, similar to the two largest TAM clusters seen in our Py8119 orthotopic model whereas TAMs arising from normal ductal macrophages, i.e. tissue resident macrophages, were only seen in early in the development of spontaneous PyMT tumors (45). Thus, similar to Py8119 tumors, almost all TAMs in larger spontaneous tumors are likely to be monocytic in origin. One notable difference between spontaneous PyMT tumors and Py8119 tumors is the homogenous expression of pro-tumoral markers, including Spp1, in each of the TAM subtypes in the orthotopic model compared to the much higher expression of Spp1 in immunoregulatory TAMs than inflammatory TAMs in the spontaneous model (**Figure 5C**) (45). It is possible that homogenous expression of pro-tumoral markers across all TAMs in the orthotopic model has prevented identification of a more deleterious subtype that could be therapeutically targeted. Nevertheless, interstitial motility is a behavior common to all TAMs, making HCK a compelling target for anti-tumoral drug development, particularly for aggressively invasive TNBCs that are known to recruit significantly higher numbers of TAMs than other, less aggressive breast cancer subtypes (22). In summary, the Py8119 model of breast cancer provides useful insights into the behavior of TAMs in TNBC, particularly HCK-dependent motile TAMs at the invasive front of these cancers. In addition, our demonstration that HCK promotes tumor growth and dissemination via TAM invasive behavior as well as by blocking CD8^+^ T cell anti-tumor immunity increases the urgency to develop highly selective HCK inhibitors for use in the clinic.

## Data Availability

The datasets presented in this study can be found in online repositories. The names of the repository/repositories and accession number(s) can be found in the article/**Supplementary Material**.

## Supplementary material

Supplementary Table 1 Antibodies used.

Supplementary Figure 1 Py8119 cell *in vitro* data. **(A, B)** Relative expression of SFKs in Py8119 cells. **(C, D)** Proliferation of Py8119 cells treated with RK20449 or dasatinib, *p < 0.05.

Supplementary Figure 2 Anti-pHck antibody is non-specific. pY HCK (red in merge) co-localizes with DAPI (blue in merge) in Py8119 tumor nuclei in *Hck^KO^* mice. Iba1^+^ TAMs are green in merge. Scale bar, 200µm.

Supplementary Figure 3 CD3^+^ T cells increase 4-fold in HCK-deficient Py8119 tumors but NK cell depletion does not significantly affect Py8119 tumor growth. **(A)** Quantification of CD3+ T cells and CD3^+^;CD8^+^ T cells as percent of live cells in *Hck^WT^* and *Hck^KO^* tumors. Data are represented as mean ± SEM and significance testing was conducted using student’s T-test. **(B)** Quantification of CD3+ T cells and CD3^+^;CD8^+^ T cells as percent of live cells in *Hck^WT^* and *Hck^CA^* tumors. Data are represented as mean ± SEM and significance testing was conducted using student’s T-test. **(C)** Survival of Py8119 tumor-bearing *Hck^WT^* (circles, black lines), *Hck^KO^* (triangles, blue lines) and *Hck^CA^* (squares, red lines) mice treated with anti-NK1.1 antibody (solid lines) or IgG isotype control (dashed lines). Mean ± SEM, *p < 0.05.

Supplementary Figure 4 TAMs in Py8119 tumors express the AXL ligand, GAS6, but not high affinity EGFR receptor ligands. **(A)** Violin plots comparing expression levels of high affinity EGFR ligands, EGF, TGFα and betacellulin (Btc) in TAMs and Py8119 cells. **(B)** CellChat-generated signaling pathway networks showing CSF-1 produced by Py8119 cells, fibroblasts and neutrophils. And GAS6 produced by TAMs and signaling to Py8119 cells and fibroblasts. **(C)** CellChat list of inferred cell-cell communication from TAMs (ligand) to Py8119 cells (receptor). **(D)** Matrigel-boyden chamber invasion assay demonstrating that Py8119 cells are not invasive in the absence of BMM or when BMM are cultured on the bottom well and not in direct contact with Py8119 cells. Data are represented as mean ± SEM, significance testing was conducted using student’s T-test. ***p < 0.001.

Supplementary Figure 5 HCK does not induce a classically activated or an alternatively activated TAM phenotype in Py8119 mammary tumors. **(A)** Violin plots comparing expression levels of classical activation markers in Py8119 tumor TAMs from control (salmon pink) or HCK mutant/inhibited (teal) mice. **(B)** Violin plots comparing expression levels of alternative activation markers in Py8119 tumor TAMs from control or HCK mutant/inhibited mice. **(C)** Pathway analysis of DEGs in TAMs from vehicle- and RK20449-treated tumors using the Enrichr biological processes gene set library.
