# Supplementary material for: The myeloid SRC family kinase HCK regulates breast cancer growth by activating tumor-associated macrophage-led invasion and inhibiting cytotoxic T cell activity
Source: Front Immunol. 2026 Feb 18;17:1709102. doi: 10.3389/fimmu.2026.1709102 (PMC12956780; doi:10.3389/fimmu.2026.1709102)
Supplement: Supplementary Table 1 — Antibodies used. [file Table1.docx]

**Supplementary Table 1.** Antibodies used

| Antibody | Clone | Host | Supplier | RRID | Dilution |
| --- | --- | --- | --- | --- | --- |
| Iba1 |  | Goat | Wako | RRID:AB_2935833 | 1:500 |
| Iba1 |  | Rabbit | Wako | RRID:AB_839504 | 1:500 |
| pTyr416 SFK | D49G4 | Rabbit | Cell Signaling | RRID:AB_10013641 | 1:100 |
| pTyr410 Hck |  | Rabbit | Abcam | RRID:AB_942255 | 1:100 |
| CD3 | 741716 | Mouse | BD Biosciences | RRID:AB_2871089 | 1:200 |
| CD8 | 755241 | Rat | BD Biosciences | RRID:AB_3687648 | 1:200 |
| CD8a | 4SM15 | Rat | Thermo Fisher | RRID:AB_2572861 | 1:200 |
| Perforin | E3W41 | Rabbit | Cell Signaling | RRID:AB_2857978 | 1:200 |
| Anti-goat IgG Ab Alexa Fluor™ 568 |  | Donkey | Thermo Fisher | RRID:AB_2534104 | 1:500 |
| Anti-goat IgG Ab Alexa Fluor™ 488 |  | Donkey | Thermo Fisher | RRID:AB_2535792 | 1:500 |
| Anti-rat IgG Ab Alexa Fluor™ 488 |  | Donkey | Thermo Fisher | RRID:AB_2535794 | 1:500 |
| Anti-rabbit IgG Ab Alexa Fluor™ 568 |  | Goat | Thermo Fisher | RRID:AB_143157 | 1:500 |
| EnVision anti rabbit/mouse |  |  | Dako | K500711-2 | N/A |
| CD8^+^ T cell depletion | YTS169 | Rat | JPP Biologics | RRID:AB_322770 | N/A |
| NK1^+^ T cell depletion | PK136 | Mouse | JPP Biologics | RRID:AB_630043 | N/A |
