# Supplementary figures and images for: The myeloid SRC family kinase HCK regulates breast cancer growth by activating tumor-associated macrophage-led invasion and inhibiting cytotoxic T cell activity
Source: Front Immunol. 2026 Feb 18;17:1709102. doi: 10.3389/fimmu.2026.1709102 (PMC12956780; doi:10.3389/fimmu.2026.1709102)

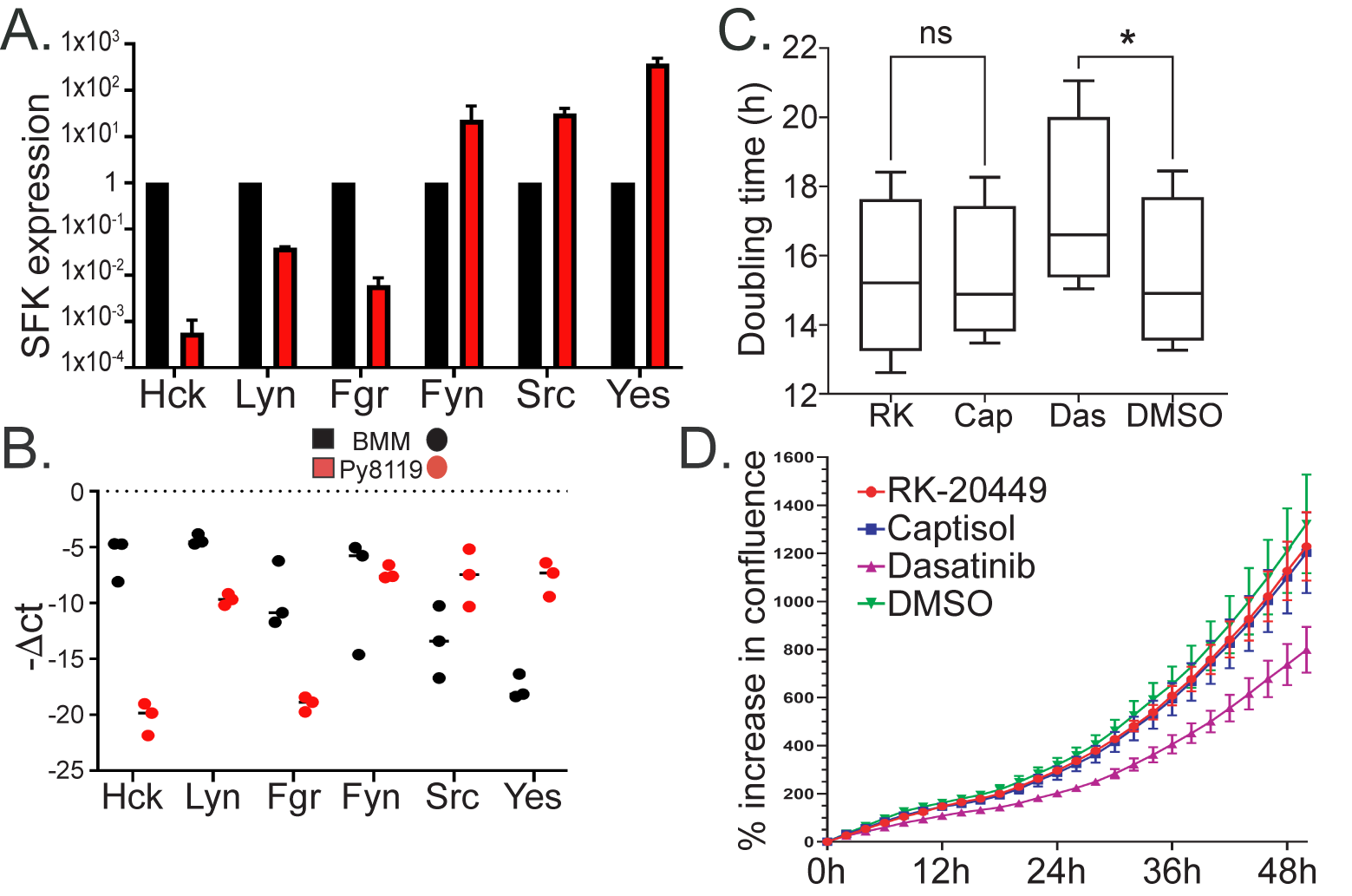

Supplement: Supplementary Figure 1 — Py8119 cell in vitro data. (A, B) Relative expression of SFKs in Py8119 cells. (C, D) Proliferation of Py8119 cells treated with RK20449 or dasatinib, *p < 0.05. [file Image1.tif]

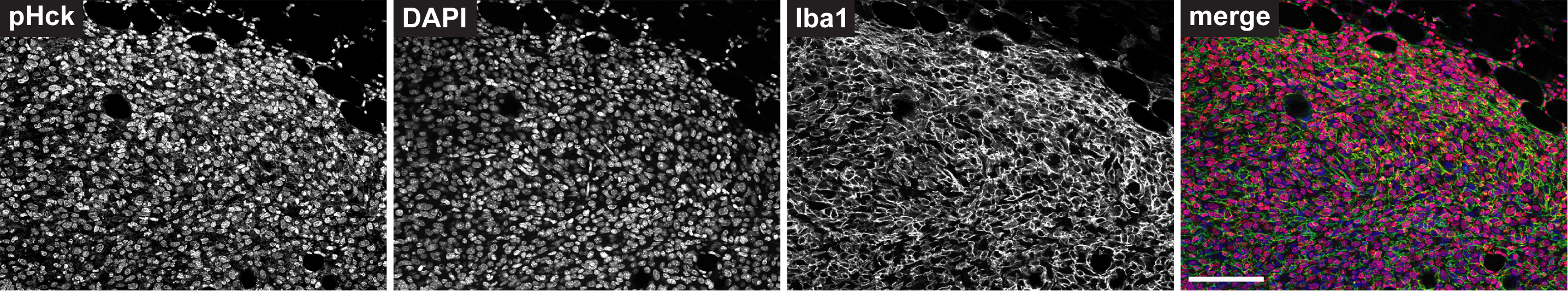

Supplement: Supplementary Figure 2 — Anti-pHck antibody is non-specific. pY HCK (red in merge) co-localizes with DAPI (blue in merge) in Py8119 tumor nuclei in HckKO mice. Iba1+ TAMs are green in merge. Scale bar, 200µm. [file Image2.tif]

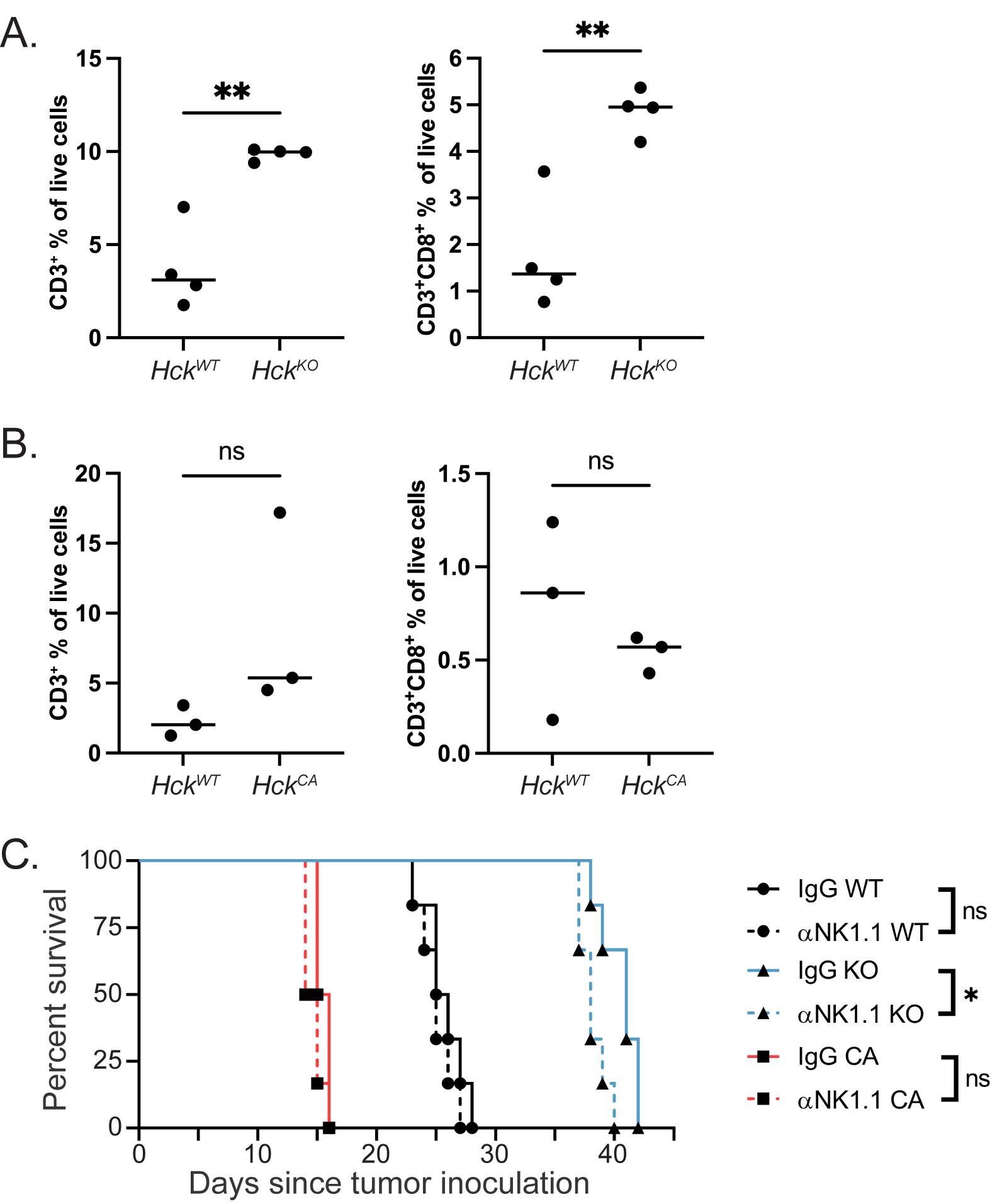

Supplement: Supplementary Figure 3 — CD3+ T cells increase 4-fold in HCK-deficient Py8119 tumors but NK cell depletion does not significantly affect Py8119 tumor growth. (A) Quantification of CD3+ T cells and CD3+;CD8+ T cells as percent of live cells in HckWT and HckKO tumors. Data are represented as mean ± SEM and significance testing was conducted using student’s T-test. (B) Quantification of CD3+ T cells and CD3+;CD8+ T cells as percent of live cells in HckWT and HckCA tumors. Data are represented as mean ± SEM and significance testing was conducted using student’s T-test. (C) Survival of Py8119 tumor-bearing HckWT (circles, black lines), HckKO (triangles, blue lines) and HckCA (squares, red lines) mice treated with anti-NK1.1 antibody (solid lines) or IgG isotype control (dashed lines). Mean ± SEM, *p < 0.05. [file Image3.tif]

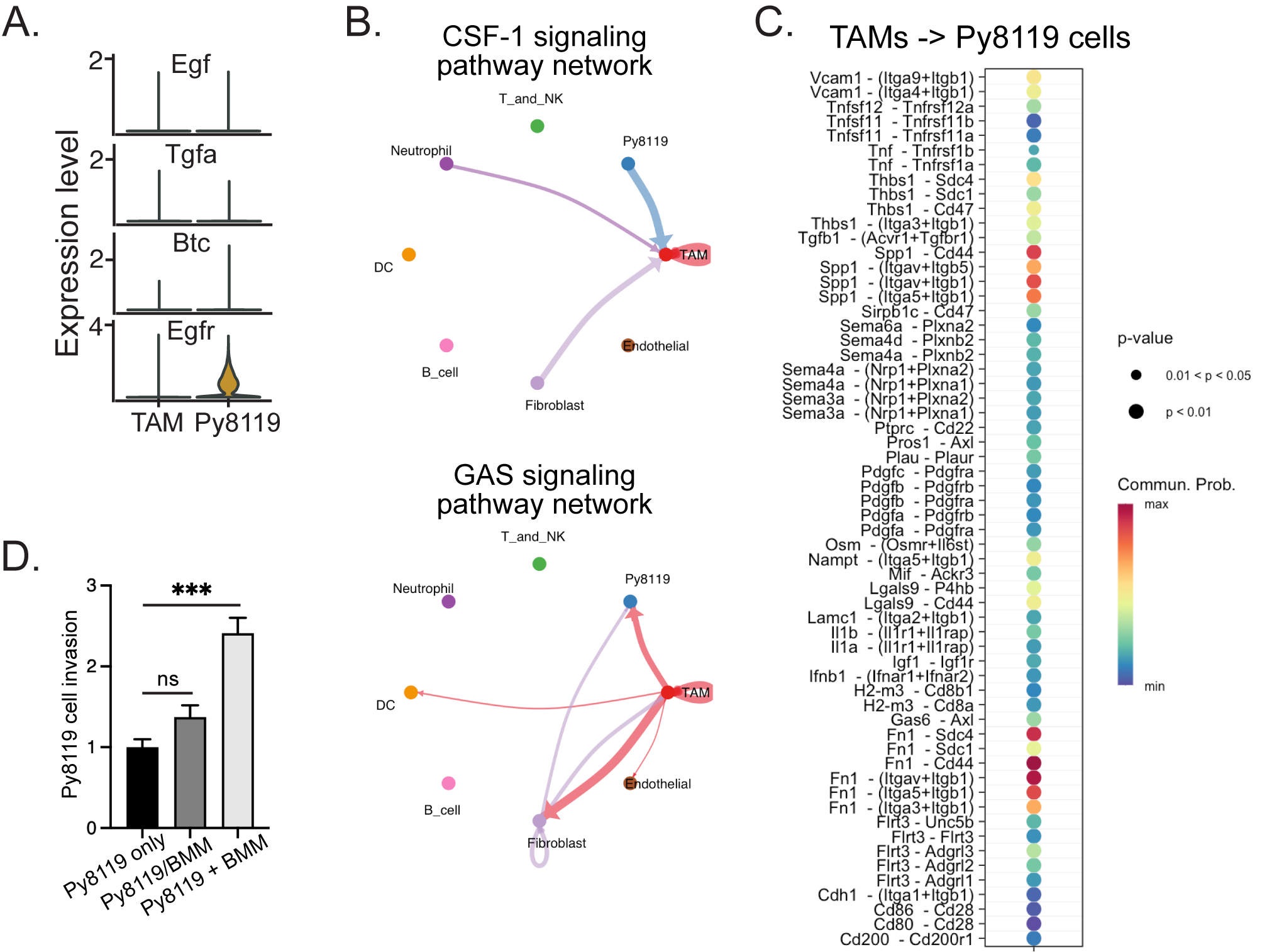

Supplement: Supplementary Figure 4 — TAMs in Py8119 tumors express the AXL ligand, GAS6, but not high affinity EGFR receptor ligands. (A) Violin plots comparing expression levels of high affinity EGFR ligands, EGF, TGFα and betacellulin (Btc) in TAMs and Py8119 cells. (B) CellChat-generated signaling pathway networks showing CSF-1 produced by Py8119 cells, fibroblasts and neutrophils. And GAS6 produced by TAMs and signaling to Py8119 cells and fibroblasts. (C) CellChat list of inferred cell-cell communication from TAMs (ligand) to Py8119 cells (receptor). (D) Matrigel-boyden chamber invasion assay demonstrating that Py8119 cells are not invasive in the absence of BMM or when BMM are cultured on the bottom well and not in direct contact with Py8119 cells. Data are represented as mean ± SEM, significance testing was conducted using student’s T-test. ***p < 0.001. [file Image4.tif]

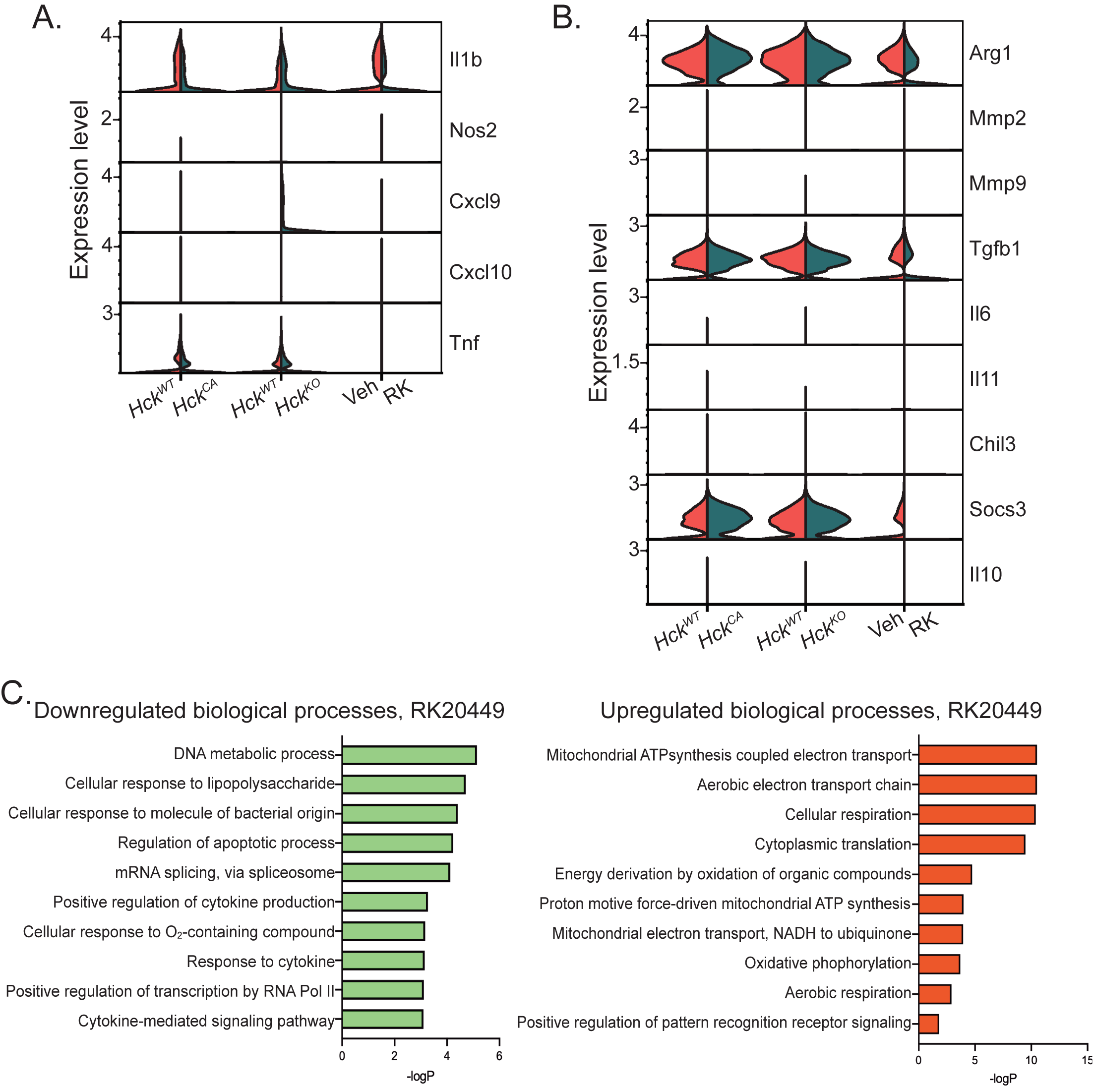

Supplement: Supplementary Figure 5 — HCK does not induce a classically activated or an alternatively activated TAM phenotype in Py8119 mammary tumors. (A) Violin plots comparing expression levels of classical activation markers in Py8119 tumor TAMs from control (salmon pink) or HCK mutant/inhibited (teal) mice. (B) Violin plots comparing expression levels of alternative activation markers in Py8119 tumor TAMs from control or HCK mutant/inhibited mice. (C) Pathway analysis of DEGs in TAMs from vehicle- and RK20449-treated tumors using the Enrichr biological processes gene set library. [file Image5.tif]
